# Diagnostic dilemmas in acute type A aortic dissection with acute myocardial infarction: A fatal misdiagnosis case and improvement strategies

**DOI:** 10.1097/MD.0000000000045809

**Published:** 2025-11-14

**Authors:** Yunyun Zhou, Han Chen

**Affiliations:** a Department of Intensive Care Unit, Wuhan Asia General Hospital, School of Medicine, Wuhan University of Science and Technology, Wuhan, China.

**Keywords:** acute myocardial infarction, acute type a aortic dissection, diagnostic pitfalls, iatrogenic injury, percutaneous coronary intervention

## Abstract

**Rationale:**

Acute type A aortic dissection (TA-AAD) is a life-threatening cardiovascular emergency, with 1% hourly mortality in untreated patients and urgent surgical intervention required upon diagnosis. However, its clinical manifestations overlap significantly with acute myocardial infarction (AMI), leading to persistently high misdiagnosis rates in practice. Existing clinical guidelines lack targeted differential protocols for coexistent TA-AAD and AMI, often resulting in misclassification (e.g., TA-AAD as AMI) and subsequent inappropriate treatment that raises mortality. Thus, case analysis is urgently needed to identify diagnostic dilemmas and optimize clinical pathways.

**Patient concerns:**

This report analyzes a patient with Stanford TA-AAD who initially presented with acute anterior myocardial infarction (a high-risk case requiring mechanical circulatory support). The patient was admitted for persistent chest pain.

**Diagnoses:**

The patient had electrocardiogram findings of coved ST-segment elevation and significantly elevated myocardial enzymes, meeting ST-segment elevation myocardial infarction criteria. After percutaneous coronary intervention, refractory low cardiac output persisted, prompting extracorporeal membrane oxygenation support. Ascending aortic dissection was accidentally detected by bedside ultrasound, confirming TA-AAD.

**Interventions:**

The patient underwent percutaneous coronary intervention and subsequent major vascular surgery.

**Outcomes:**

Despite surgical intervention, the patient ultimately died.

**Lessons:**

This case underscores the need for clinicians to be alert to the coexistence of TA-AAD and AMI in chest pain patients: percutaneous coronary intervention may exacerbate dissection progression and surgical hemostatic difficulty, while misdiagnosis combined with inappropriate treatment markedly increases mortality. Analyzing this fatal misdiagnosis provides practical references for optimizing diagnostic/therapeutic workflows, promoting multidisciplinary team involvement in decision-making, and reducing misdiagnosis-related adverse outcomes – ultimately offering valuable clinical guidance for improving patient survival.

## 1. Introduction

TA-AAD is a life-threatening cardiovascular emergency, with an hourly mortality rate of 1% in untreated patients^[1]^; immediate surgical intervention is required upon diagnosis.^[2,3]^ Despite ongoing advancements in surgical techniques and perioperative monitoring, diagnostic pitfalls remain when TA-AAD presents as AMI, often leading to fatal management errors.^[4,5]^ This case report describes a fatal TA-AAD patient initially diagnosed with ST-segment elevation myocardial infarction (STEMI), whose aortic dissection progression was accelerated following PCI. Retrospective analysis of this case reveals 2 key issues in current STEMI management workflows: the neglect of aortic lesion screening, and the unrecognized hazardous interaction between antithrombotic therapy and occult dissection. Accordingly, this report aims to highlight the inadequacy of aortic lesion assessment in existing STEMI protocols, clarify the potential risks of antithrombotic therapy in undiagnosed TA-AAD patients, and propose a time-sensitive diagnostic protocol that integrates detailed physical examination, D-dimer testing, and imaging studies, aiming to prevent the occurrence of iatrogenic injury.

## 2. Case report

**Figure 1.**
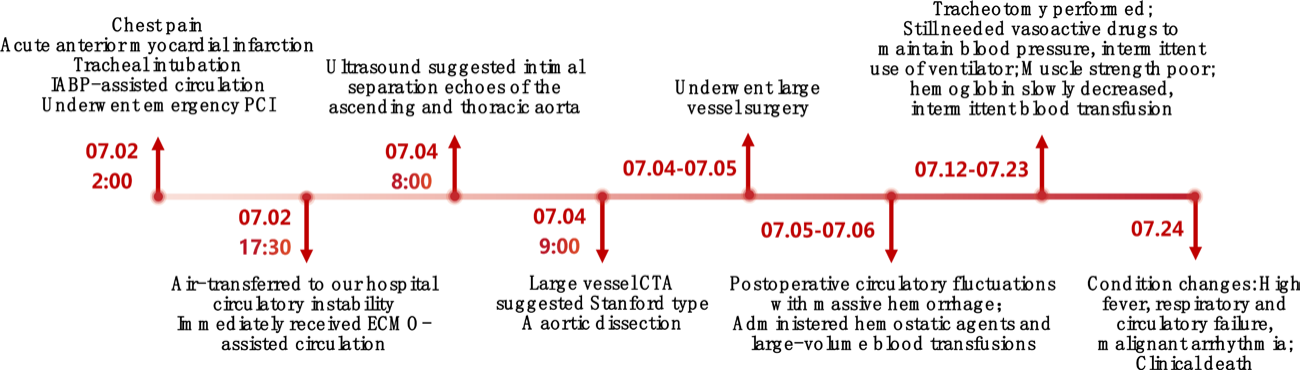
Flow chart of the disease course.

A 52-year-old male patient (height 178 cm, weight 90 kg) with a 10-year history of hypertension was referred to our hospital for further management. His hypertension was controlled with oral medications, with a maximum recorded blood pressure of 160/120 mm Hg. He had no previous surgical history or relevant family history. The patient was admitted to another hospital at 2:00 a.m. on July 2 due to sudden onset of chest pain. Based on initial findings at that hospital-including ST-segment elevation on electrocardiography (suggestive of acute anterior myocardial infarction, STEMI) and severe circulatory instability with respiratory failure-he was given immediate endotracheal intubation, followed by emergency coronary stent implantation under intra-aortic balloon pump (IABP)-assisted circulation. Despite post-procedural administration of high-dose vasoactive drugs, the patient’s circulation remained unstable. He was then transferred to our hospital by air ambulance for further optimized treatment. (The specific flow chart of the disease course is shown in Fig. 1.)

**Figure 2.**
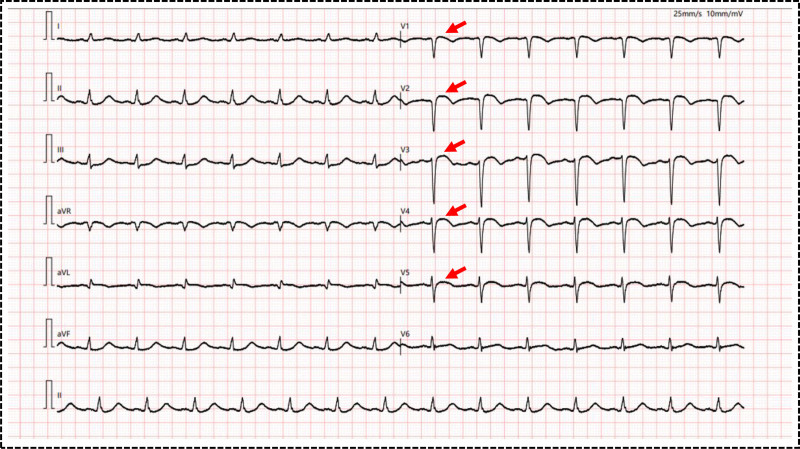
Twelve-leads ECG shows ST-segment elevation in leads V1-V5, indicating acute anterior myocardial infarction. ECG = electrocardiogram.

Upon admission to our hospital, relevant examinations completed showed the following results: white blood cell count 22.04 × 10⁹/L, plasma D-dimer 18.55 mg/L, serum creatinine 270 μmol/L, high-sensitivity troponin T 40833 ng/L, myoglobin 4356 ng/mL, and N-terminal pro-B-type natriuretic peptide (NT-proBNP) 5918.0 pg/mL. No significant abnormalities were found in other laboratory indicators. Emergency bedside echocardiography confirmed acute anterior myocardial infarction (Fig. 2) and showed impaired systolic function of both ventricles, with left and right ventricular ejection fractions reduced to 30% and 32%, respectively. The patient had severe cardiogenic shock. Additionally, emergency bedside echocardiography revealed poor cardiac systolic function. As circulatory stability could not be maintained even with vasoactive drugs and IABP support, extracorporeal membrane oxygenation was immediately initiated for circulatory support.

**Figure 3.**
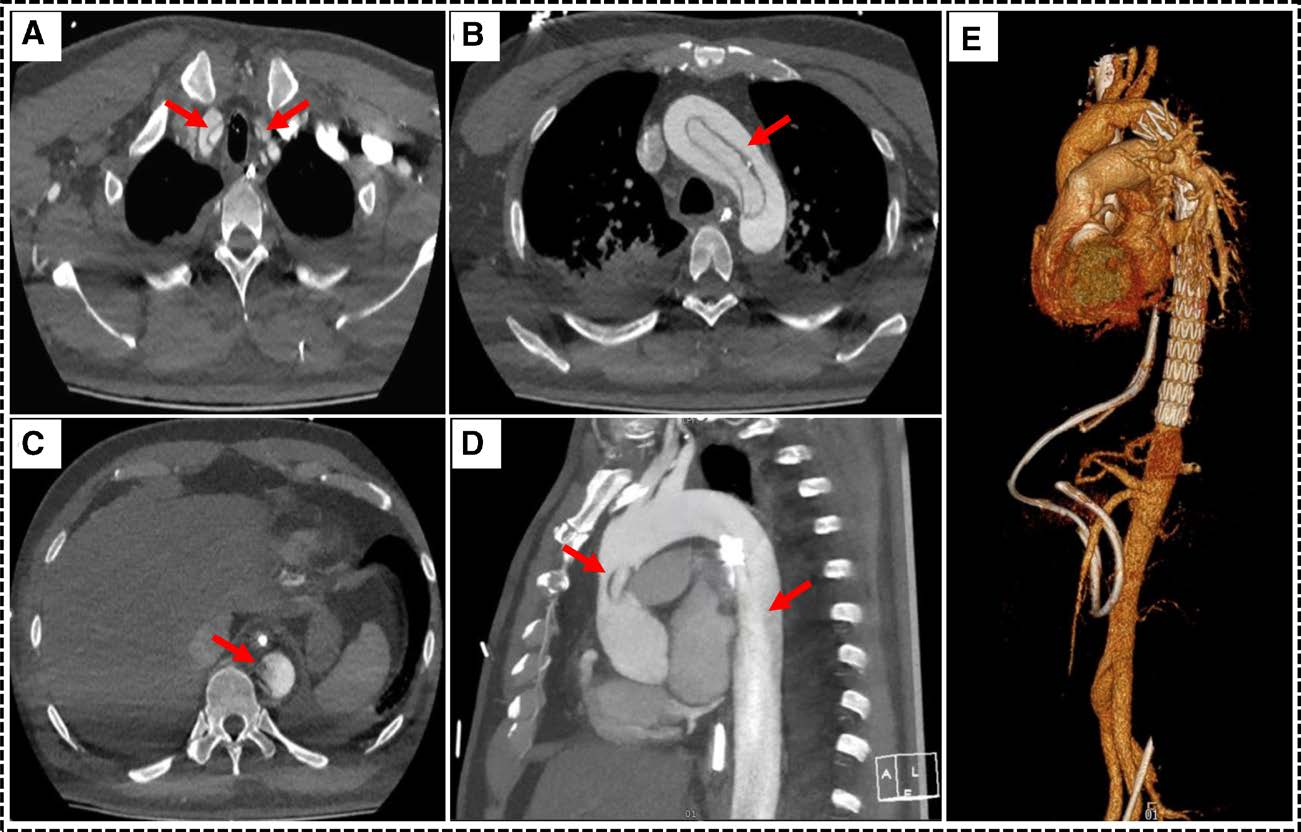
Preoperative vascular CTA. (A) Involvement into the innominate artery and the left common carotid artery (red arrows); (B) Involvement into the aortic arch (red arrows); (C) Involvement into the abdominal aorta, and the small-sized true-lumen (red arrows); (D) Involvement into the entire aorta (red arrows); (E) Three-dimensional reconstruction after type A aortic dissection surgery. CTA = computed tomography angiography.

On July 4, a comprehensive great vessel echocardiography showed intimal dissection echoes in the ascending aorta and thoracic aorta, suggesting a possible aortic dissection. Subsequent computed tomography angiography (CTA) of the great vessels confirmed the diagnosis of Stanford Type A aortic dissection, which involves the right innominate artery, bilateral common carotid arteries, both coronary arteries and the celiac trunk artery (Fig. 3a–d). Given the potential risk of IABP exacerbating aortic dissection progression, the IABP was removed at the same day and the patient immediately underwent emergency procedures including thoracic aortography, thoracic aortic stent implantation, Bentall procedure (aortic valve replacement, ascending aortic replacement, and coronary artery bypass grafting), total aortic arch replacement with stented elephant trunk implantation (Sun’s procedure), and intraoperative cardiac pacemaker implantation (Fig. 3e).

Postoperatively, the patient had significant circulatory fluctuations and required high-dose vasoactive drugs to maintain blood pressure, with the specific regimen as follows: norepinephrine at 0.7 μg/kg/min, dobutamine at 5 μg/kg/min, epinephrine at 0.05 μg/kg/min, and posterior pituitary hormone at 2 U/h. Meanwhile, the patient experienced massive postoperative bleeding, for which pharmacologic hemostatic therapy and massive blood transfusion were administered. Despite a period of postoperative treatment, the patient still required vasopressors to maintain blood pressure and received anti-infective treatment. He had difficulty in weaning from the ventilator and underwent tracheostomy, with intermittent successful weaning attempts. Despite adjustments in treatment, vasopressors could not be discontinued. The patient developed gastrointestinal bleeding, and hemoglobin levels dropped, necessitating the cessation of antiplatelet therapy. On the night of July 24, the patient presented with the symptom of high fever, respiratory distress, and significant circulatory instability. The antipyretics were immediately used to bring down the fever. Despite treatment with fluids, and vasopressors, his blood pressure dropped sharply to 50/30 mm Hg. Immediately, cardiopulmonary resuscitation and pharmacological resuscitation were initiated. The bedside ultrasound examination indicated ventricular asynergy and electromechanical dissociation. Finally, the patient suffered cardiac arrest and died.

Notably, 2 primary causes of misdiagnosis were identified in this case. First, aortic lesion screening was neglected in the early diagnostic process-this step is critical for differentiating aortic dissection from AMI, and its omission laid the groundwork for initial diagnostic bias. Second, timely imaging examinations (such as CTA) were not performed, resulting in failure to identify the etiological cause. The initial onset of the condition might have been attributed to aortic dissection involving the coronary arteries; alternatively, it could have stemmed from iatrogenic dissection induced during PCI. Owing to the absence of this critical differential diagnostic tool, the patient was misdiagnosed as having STEMI in the early stage of the disease.

## 3. Discussion

The patient in this case presented with chest pain as the main clinical manifestation, accompanied by coved ST-segment elevation on electrocardiogram and elevated myocardial enzyme levels. Notably, aortic dissection involving the coronary arteries can also present with similar symptoms, leading to significant challenges in clinical differentiation. To enhance diagnostic accuracy, the current diagnosis and treatment workflow requires further optimization. Specifically, routine 4-limb blood pressure monitoring should be implemented to assess for the presence of blood pressure asymmetry; chest radiography should be completed to evaluate for indicators including pericardial effusion, pleural effusion, mediastinal widening, and abnormalities in aortic contour or structure; and comprehensive color Doppler ultrasonography should be performed for routine assessment of key parameters such as pericardial effusion, aortic valve regurgitation, aortic diameter, and the presence of intimal flaps in large blood vessels. Additionally, if a patient presents with an elevated D-dimer level, the possibility of aortic dissection must be prioritized in the differential diagnosis.

In current clinical practice, revascularization therapy for patients with acute STEMI is administered in a relatively timely manner. However, incorporating great vessel ultrasound and great vessel CTA into the diagnostic workflow before PCI can significantly reduce the misdiagnosis rate. In this case, there was a risk of iatrogenic injury during the PCI procedure. Further great vessel CTA examination revealed that the dissecting lesion involved both coronary arteries, with a localized hypodense area near the left coronary artery ostium adjacent to the aortic sinus, which was considered a hematoma. The patient received dual antiplatelet loading therapy before PCI and heparin anticoagulation during the procedure, which significantly increased the difficulty of hemostasis in subsequent major vascular surgery.

Postoperatively, the patient experienced circulatory fluctuations and massive bleeding, which further exacerbated disease progression. Additionally, the patient’s initial etiology was not clarified, and coupled with inappropriate sequential interventions, these factors collectively contributed to further deterioration of the patient’s condition. Meanwhile, the patient had severe cardiac dysfunction and required vasoactive drugs to maintain stable blood pressure. During the disease course, the patient developed gastrointestinal bleeding, prompting the discontinuation of antiplatelet therapy. Concurrently, the patient presented with high fever and septic shock; despite antipyretic treatment, massive sweating led to a further reduction in effective circulating blood volume, resulting in hypotension and inadequate coronary perfusion, which further aggravated coronary ischemia. Previous studies have confirmed that the mortality rate of STEMI patients complicated with cardiogenic shock is significantly higher.^[6,7]^ Eventually, the patient experienced a sudden deterioration in condition: a decrease in heart rate was followed by a sharp drop in blood pressure, and even with the gradual increase in the dose of vasoactive drugs, blood pressure could not be maintained stably. Subsequent malignant arrhythmia and severe deterioration of cardiac function ultimately led to the patient’s death.

Several clinical insights can be derived from this case. To begin with, it is essential to abandon the mindset that “chest pain equates to myocardial infarction.” Instead, thorough physical examinations should be conducted, 4-limb blood pressure measured, and basic examinations including ultrasonography, chest radiography, and D-dimer testing completed. For patients suspected of aortic dissection, further “triple rule-out” CT scans (i.e., ruling out aortic dissection, pulmonary embolism, and myocardial infarction via CTA) are recommended. Additionally, great vessel ultrasound and the aforementioned triple rule-out CT for chest pain should be integrated into the core diagnostic workflow for chest pain. Next, a multidisciplinary collaborative diagnosis and treatment model should be established, where cardiologists, cardiac surgeons, radiologists, and intensivists jointly participate in formulating treatment decisions. Furthermore, it is advisable to develop an artificial intelligence (AI)-assisted early warning model to enable early risk stratification and warning for high-risk patients presenting with chest pain.

Although this case analysis identifies key issues and optimization directions in TA-AAD complicated with AMI management and provides clinical references, it has limitations that require objective consideration of the scope of its conclusions. To begin with, the case first suffers from insufficient sample representativeness and incomplete follow-up. Only one AMI patient with TA-AAD who underwent PCI was included, and the uniqueness of this specific case creates barriers to extrapolating the research conclusions to a broader population of similar patients. Moreover, the patient survived merely 22 days from admission to death. The lack of long-term follow-up data-including CTA and cardiac function assessments at 1 to 3 months postoperatively-precludes the evaluation of the long-term prognostic impact of the applied diagnosis and treatment strategy. Additionally, as a retrospective case report, the integrity of the data is heavily dependent on the quality of original medical records. Existing deficiencies in documentation-including missing descriptions of symptom evolution (e.g., changes in the nature or location of pain), ambiguous time nodes for imaging examinations, and incomplete data from prior care at other hospitals directly compromise the accuracy of analyzing the causes of misdiagnosis, introducing potential bias into the attribution of clinical errors.

Beyond that, the results of this case report are also limited by the diagnostic and treatment conditions specific to the institution, including variations in emergency differential diagnosis workflows, discrepancies in the resolution of imaging equipment, and differences in the experience level of the cardiac care team. These factors collectively undermine the generalizability of the conclusions. Another key limitation lies in the insufficient depth of technical analysis. While this case report identifies ST-T segment changes on the electrocardiogram as the primary cause of misdiagnosis, it fails to establish a multimodal differential pathway-it lacks a standardized diagnostic and treatment process that integrates physical examinations, biomarker testing (e.g., D-dimer), and imaging studies (chest X-rays, ultrasound, and CTA).

## 4. Conclusion

Misdiagnosing Stanford TA-AAD as STEMI can lead to catastrophic consequences. To address this issue, the diagnostic workflow must be optimized through 3 pivotal measures: standardized screening (conducting detailed physical examinations, completing chest radiography, ultrasonography, D-dimer testing, and other relevant examinations, with triple-rule-out CTA being performed when necessary), multidisciplinary collaboration, and AI-based early warning systems. These strategies aim to achieve early differential diagnosis, secure the critical window for rescue treatment, and ultimately improve patient prognosis.

## Author contributions

**Conceptualization:** Yunyun Zhou.

**Data curation:** Yunyun Zhou.

**Investigation:** Yunyun Zhou, Han Chen.

**Methodology:** Yunyun Zhou, Han Chen.

**Validation:** Yunyun Zhou.

**Writing – original draft:** Yunyun Zhou.

**Writing – review & editing:** Yunyun Zhou.
